# COVID-19: an update and cardiac involvement

**DOI:** 10.1186/s13019-020-01299-5

**Published:** 2020-09-09

**Authors:** Nizar R. Alwaqfi, Khalid S. Ibrahim

**Affiliations:** Princess Muna Heart Centre, Department of General Surgery, Faculty of Medicine, Jordan University of Science and Technology and King Abdullah University Hospital, Floor 8C, P O Box 3030, Irbid, 22110 Jordan

**Keywords:** COVID-19, SARS-CoV-2, Cardiac involvement

## Abstract

Severe acute respiratory syndrome coronavirus 2 (SARS-CoV-2) infects host cells through angiotensin converting enzyme 2 receptors, leading to coronavirus disease (COVID-19)-related pneumonia, and also causing acute cardiac injury and chronic damage to the cardiovascular system. The purpose of this review is primarily reviewing the COVID-19 disease, including pathogen, clinical features, diagnosis, and treatment with particular attention to cardiovascular involvement based on the current evidence. COVID-19 remains a threat to global public health. The associated extra-pulmonary manifestations and their prolonged consequences are frequently overlooked. Pre-existing cardiovascular disease or acute cardiac complications may contribute to adverse early clinical outcome. At the moment, there is no specific treatment for COVID-19, but multiple randomized controlled trials (RCT) are being conducted. New supportive therapies are being evaluated with promising results.

## Background

In the last two decades the family coronaviruses (CoVs) was responsible for two severe epidemics of zoonotic origin. In 2003 a mysterious pneumonia, originated from southeast China, caused by a new CoV and was named severe acute respiratory syndrome CoV (SARS-CoV), it infected more than 8000 with a mortality rate around 10–15% with no available proper treatment or vaccination. Then emergence of another outbreak in 2012 in the Middle East of a novel CoV called Middle East respiratory syndrome CoV (MERS-CoV), it infected 857 cases with 35% mortality rate [1–3].

In late December 2019, an outbreak of a mysterious pneumonia happened in a seafood wholesale wet market, the Huanan Seafood Wholesale Market, in Wuhan, Hubei, China [4, 5]. The underlying causative agent of this outbreak was identified as a novel coronavirus, that was named severe acute respiratory syndrome CoV 2 (SARS-CoV-2) and the disease related to it as CoV disease 2019 (COVID-19) by the World Health Organization (WHO). Later, WHO named this pathogenic virus for 2019-nCoV [6, 7]. The market was closed on 1 January 2020 [5]. SARS-CoV-2 genetic sequence was shared publicly on 11–12 January, it is an enveloped virus with a genetic material made up of a positive–sense single–stranded RNA [5, 8]. On march 11, 2020 WHO declared COVID-19 a pandemic disease and by May 12, 2020 the virus has spread to more than 200 countries worldwide with more than 4 million cases and more than 283 thousand deaths [5]. Till now, all available evidence for COVID-19 suggests that SARS-CoV-2 has a zoonotic origin in bats and not a laboratory construct [5].

Many literature reported the clinical features, virology, pathophysiology, epidemiology, and radiology of COVID-19, but the comprehensive review is few. And although COVID-19 is predominantly a respiratory disease, some patients develop severe cardiovascular disease [1]. In addition, patients with underlying cardiovascular disease might have an increased risk of mortality [1]. The purpose of this review is to summarize the current literature on COVID-19 disease, including pathogen, clinical features, diagnosis, and treatment based on the current evidence, with emphasis on understanding the mechanisms of cardiac involvement, cardiac complications, so that treatment of these patients can be timely and effective and mortality reduced.

### Virology and methods of transmission

Coronavirus is an enveloped, positive-sense single-strand RNA virus. It is classified within the Orthocoronavirinae subfamily, with the characteristic “crown-like” spikes on their surfaces [6]. The SARS-CoV, bat SARS-like CoV and others fall into the genus beta-coronavirus [9]. The genus beta-coronavirus can be divided into several subgroups. The SARS-CoV-2, SARS-CoV, and bat SARS-like CoV belong to Sarbecovirus, while the MERS-CoV to Merbecovirus [4]. SARS-CoV, MERS-CoV, and 2019-nCoV all cause diseases in humans but each subgroup may have mild different biologic characteristic and virulence [6–10].

Because of the sequence identity of the bat-CoV, it is likely the SARS-CoV-2 originated in bats, however the intermediate host between bat reservoir and human is still unclear [4, 11, 12].

According to current evidence, human to human transmission of COVID-19 virus is either direct through respiratory droplets or indirect through contact routes by touching objects contaminated by others then touching nose, mouth, and eyes [13–17]. In an analysis of 75,465 COVID-19 cases in China, airborne transmission was not reported [18]. But to date, some scientific publications provide initial evidence on whether the COVID-19 virus can be detected in the air [19, 20], which has led many countries to recommend following appropriate precautions. Bahl P et al. [ 20], concluded that weight of combined evidence supports airborne or droplet precautions for health workers treating COVID-19. Although there is some evidence that SARS-CoV-2 can be cultured from stool of infected persons, however, to date disease transmission through the faecal-oral route is unknown [21].

The COVID-19 virus is highly contagious, it is estimated that the reproductive number (RO) (i.e. patient’s capability to spread the disease to people in contact) in a range of 2.20–3.58 [13, 22, 23]. A key factor in the transmissibility of Covid-19 is the high level of SARS-CoV-2 shedding in the upper respiratory tract, even among asymptomatic patients, which distinguishes it from SARS-CoV, where replication occurs mainly in the lower respiratory tract [24, 25].

The SARS-CoV-2 can survive on different surfaces in the environment that varies from few hours to as long as 7 days, depending on surface type [26]. The virus is highly stable at 4 °C, but at a temperature of 70 °C, the time for virus inactivation is 5 min [26].

### Cardiac involvement

COVID-19 is an acute disease that primarily targets the respiratory system. 2019-nCOV uses its S-spike to bind angiotensin converting enzyme (ACE)2 receptors as an entry point to the cell. ACE2 receptor expressed in both type1 and type 2 pneumocytes but also expressed in other types of cells, including endothelial cells [25].

Patients with cardiac risk factors and established cardiovascular disease (CVD) seem to be at an increased risk to develop COVID-19 with more severe disease and worse clinical outcomes [27, 28]. A lso acute cardiac complications caused by infection with the SARS-CoV-2, increases the difficulty and complexity of patient treatment. Thus recognizing patients with previous cardiac disease or developed cardiac complications during course of the disease, is of utmost significance due to the adverse impact on outcome [4, 28, 29]. The mechanism of myocardial injury can be any of the following:

Direct myocardial cells injury where the SARS-CoV-2 uses ACE2 receptors as an entry point to the cell. This interaction of SARS-CoV-2 with ACE2 can result in changes of ACE2 pathways leading to acute injury to the lung, heart, and endothelial cells [ 29].

Severe COVID-19 disease associated elevated levels of proinflammatory cytokines can result in injury to multiple organs, including cardiac myocytes [ 29].

Acute plaque rupture as a part of systemic inflammation and catecholamine surge inherent in this disease can lead to increased plaque vulnerability and possible rupture leading to acute coronary syndrome [29].

Myocardial oxygen supply/demand mismatch due to an increased cardiometabolic demand associated with the systemic infection, and the resultant hypoxia due to severe pneumonia or acute respiratory distress syndrome leading to inadequate supply, thus resulting in myocardial damage [29].

As a part of any critical illness, potassium and other electrolyte disturbances can lead to serious arrhythmias especially in patients with cardiac disease. Hypokalemia induced serious arrhythmias is of particular interest because of the SARS-CoV-2 interaction with renin-angiotensin system.

There are other injurious possibilities, including medications side effects of corticosteroids, antiviral medications, and immunological agents [29].

### Clinical features

The current official estimated incubation period for the SARS-CoV-2 is 2–14 days with a mean duration of 5.2 days, one asymptomatic carrier reported with an incubation period of 19 days, and almost all patients are likely to experience one or more symptoms within 12.5 days of contact [15, 23, 30]. A higher risk of infection has been noticed in older patients, male sex, patients with chronic pulmonary, chronic cardiac or chronic kidney disease, and patients with diabetes mellitus, hypertension and malignancy [31].

Patients at any age can contract infection with the SARS-CoV-2 and any system can be involved. Patients can be either asymptomatic carriers or develop symptoms, ranging from the common mild symptoms of upper respiratory tract infection of fever, dry cough, dyspnea, myalgia, and fatigue to the severe respiratory or multi-organ failure. Less common symptoms include anosmia, diarrhea, abdominal pain, dizziness, headache and confusion, and hemoptysis. Although pneumonia is present in most SARS-CoV-2 infected patients, few cases complained of pleuritic chest pain [15, 25, 27, 32].

There are four clinical classifications of patients according to Diagnosis and Treatment Protocol for Novel Coronavirus Pneumonia (Trial Version 6) that was released by the National Health Commission & State Administration of Traditional Chinese Medicine; mild, moderate, severe and critical. Patients in the mild group have mild symptoms with no radiologic findings of pneumonia. Those in the moderate group show fever, and respiratory symptoms, with radiological findings of pneumonia. Severe cases showing any of these criteria: respiratory distress (respiratory rate ≥ 30/min), hypoxemia (blood oxygen saturation ≤ 93%), and lung infiltrates progression > 50% within 24–48 h. Critical cases meeting any of the following criteria: Respiratory failure and requiring mechanical ventilation, shock, with other organ failure that requires intensive care unit (ICU) care [27]. If the disease progresses, the median duration period from illness onset to dyspnea is 8.0 days, and to mechanical ventilation is 10.5 days [4].

Although it is early to estimate COVID-19 mortality rates (MR), a large cases study indicated that the MR is elevated among patients with coexisting medical conditions [27]. WHO as of march 3, 2020 reported 3.4% global MR.

### Evaluation

When treating patients with the diagnosis of acute viral respiratory tract infection many differential diagnoses related to atypical pathogens and common viral pneumonia should be considered, such as influenza, parainfluenza, adenovirus, respiratory syncytial virus infection, etc. [4] Therefore, it is crucial to trace the travel and exposure history when approaching a suspected patient back from an epidemic area.

Common laboratory findings in patients with COVID-19 include leucopenia, and lymphopenia [4, 15, 31, 33]. Lymphopenia is a cardinal feature of COVID-19. Less common blood abnormalities may include thrombocytopenia, anemia, abnormal liver and kidney function, elevated creatine kinase and prothrombin time, increased lactate dehydrogenase and D-dimer. Inflammatory markers like serum ferritin and C-reactive protein can be elevated. Patients with cardiac involvement, troponin and brain natriuretic peptide can be elevated also [4, 15, 31, 33]. Considering patients’ and laboratory safety, physicians should carefully evaluate the necessity of frequent blood sampling and conduct aspiration to prevent the risk of unexpected exposure. Huang C. et al. report a comparison between intensive care unit (ICU) and non-ICU patients, that plasma concentrations of interleukin (IL) 2, IL7, IL10, granulocyte colony-stimulating factor (GCSF), interferon (IP)10, and tumor necrosis factor (TNF)-α are higher in ICU patients than non-ICU patients [4]**.** These findings suggest that initiation of the immune response results in the production of chemokines and cytokines, which damage normal host lung. New studies are evaluating potential abnormal coagulation cascade in severe COVID-19 cases that may lead to microthrombi in many end organs. In those patients, high D-dimer is associated with poor prognosis and a high mortality rate [34]. Computed tomography (CT) imaging of the chest in patients with COVID-19 is not specific and may overlap with other infections. In contrast to what was believed, the current recommendation of many learned societies and professional radiological associations is that imaging should not be employed as a screening/diagnostic tool for COVID-19, but reserved for the evaluation of complications [35]. According to a Fleischner Society consensus statement published on 7 April 2020: Imaging is not indicated in patients with suspected COVID-19 and mild clinical features unless they are at risk for disease progression. Imaging is indicated in a patient with COVID-19 and worsening respiratory status. In a resource-constrained environment, imaging is indicated for medical triage of patients with suspected COVID-19 who present with moderate-severe clinical features and a high pretest probability of disease [36].

Transthoracic echocardiography is recommended for inpatients with heart failure, arrhythmia, ECG changes, or newly diagnosed cardiomegaly on chest x-ray or CT-chest. However, most radiology societies have warned undue CT/MRI imaging again for cardiovascular disease purposes as it can lead to unwanted exposure of the radiology staff. Appropriate prudence is thus warranted [35].

### Diagnosis

Detection of viral RNA using real-time reverse transcriptase-polymerase chain reaction (rRT-PCR) is used to confirm the clinical diagnosis. For the most sensitive detection of 2019-nCoV, bronchoalveolar lavage fluid specimens have the highest positive detection rate of 93%, followed by sputum 72%, and nasal swabs detection rate is 63%. Both fecal and blood specimens have positive rates in only 29 and 1% of diseased patients. Regardless the high detection rates in bronchoalveolar lavage fluid, the Center for Disease Control and Prevention (CDC) in the United States recommends collecting only the upper respiratory nasopharyngeal swab to decrease the risk of aerosol droplets exposure to healthcare workers [21, 24].

### Cardiac complications

Myocardial damage caused by infection with the SARS-CoV-2 undoubtedly increases the difficulty and complexity of patient treatment. The reported common Cardiac complications of COVID-19 include; new or worsening heart failure (due to cardiomyopathy or right-sided heart failure caused by pulmonary hypertension), myocarditis, and arrhythmia. Myocardial injury associated with the SARS-CoV-2 occurred in 5 of the first 41 patients diagnosed with COVID-19 in Wuhan, which mainly manifested as an increase in hs-cTnI levels (> 28 pg/ml)^4^ In this study, four of five patients with myocardial injury were admitted to the intensive-care unit (ICU), which indicates the serious nature of the myocardial injury in patients with COVID-19. Blood pressure levels were significantly higher in patients treated in the ICU than in those not treated in the ICU [4]. In a report of 138 patients with COVID-19 disease, 7.2% had acute cardiac injury, and 16.7% had arrhythmia [31]. In another cohort of 191 patients with COVID-19, 17.2% of patients had cardiac injury [37]. A meta-analysis of six published studies from China, reported 8% of the patients had cardiac injury [28]. In a study of 416 patients with COVID-19, Cardiac injury occurred in 19.7% [38]. Thus recognizing patients with previous cardiac disease or developed cardiac complications during course of the disease, is of utmost significance due to the adverse impact on outcome [4, 28, 29].

### Treatment

Currently, there is no validated treatment for COVID-19. Due to the emerging nature of COVID-19 and the urgency of management, no randomized controlled trials are showing the efficacy of a specific treatment of the 2019-nCOV. The main strategies are symptomatic and supportive care, and to minimize complications.

Non-steroidal anti-inflammatory drugs (NSAIDs), including ibuprofen use, have been reported with clinical deterioration in some patients with severe COVID-19. The current recommendation is to avoid NSAIDs use in patients with COVID-19 disease [39].

Using corticosteroids in patients with COVID-19 can be harmful, steroids can cause fluid retention, electrolyte derangement, and hypertension and in general, because of potential harm, as was learned from SARS data that indicated an increase of viral shedding [40]. However, treatment with methylprednisolone may be beneficial for patients who develop acute respiratory distress syndrome (ARDS) [41].

New studies are evaluating potential abnormal coagulation cascade in severe COVID-19 cases that may lead to microthrombi in many end organs especially the lungs. In those patients, high D-dimer is associated with poor prognosis and a high mortality rate. Tang, N. et al., report a reduction of 28-day mortality in patients with high sepsis-induced coagulopathy (SIC) score greater than 4, or D-dimer more than 6 fold of upper limit of normal, prescribed 40–60 mg enoxaparin/day or unfractionated heparin 10,000–15,000 U/day [34].

Previously known antiviral and other drugs have been tried on patients without appropriate trials forced by overloaded sick patients and death in confirmed cases with COVID-19 disease. Multiple studies have currently been conducted investigating the potential cure effect, including hydroxychloroquine in combination with azithromycin, immune therapy, remdesivir, different antiviral medications, and the use of convalescent plasma of cured patients with COVID-19 [42–52]. Some of these drugs exhibit a potential of serious cardiac side effects, treating physicians must be aware of.

Chloroquine and hydroxychloroquine, which is the more potent synthetic form of chloroquine, is being used along with azithromycin, a macrolide antibiotic, for empirical management of COVID-19 based on a small French trial [42, 43]. Chloroquine can cause atrioventricular (AV) block and, along with azithromycin, prolonged QT interval. Hydroxychloroquine can similarly produce conduction problems in the heart. Additive effects of concomitant use of beta-blockers or calcium channel blockers can induce severe bradycardia leading to cerebral hypoperfusion with syncope [47]. The current recommendation is to assess QT before starting treatment and close monitoring in patients with additional risk factors or patients using other medications that could enhance the QT prolongation [49]. In addition to close electrolytes monitoring particularly hypokalemia, because of 2019-nCoV interaction with renin-angiotensin-aldosterone system [49]. A recent multicenter, open-label, randomized controlled trial (RCT) of hydroxychloroquine involving 150 adults admitted to hospital for COVID-19 reported no significant effect of the drug on accelerating viral clearance [50].

Lopinavir/ritonavir are protease inhibitors that are approved for HIV-1 infection treatment. Prolongation of PR and QT intervals leading to high-grade AV blocks and rarely torsade de Pointes, are reported side effects. They can decrease serum concentration of the active metabolites of clopidogrel, prasugrel while increasing that of ticagrelor as well as increasing statins levels with risk of rhabdomyolysis. Lopinavir/ritonavir potentiates the effects of factor Xa inhibitors such as apixaban and rivaroxaban through the inhibition of CYP3A4, thereby increasing bleeding risk [44]. Cao B, et al. conducted a randomized, controlled, open-label trial involving hospitalized adult patients with confirmed severe COVID-19 disease. Researchers observed no survival benefit following treatment with lopinavir/ritonavir in hospitalized adult patients with severe COVID-19 beyond standard care. Future trials in patients with severe illness may help to confirm or exclude the possibility of a treatment benefit [51].

Remdesivir is a monophosphoramidate prodrug of an adenosine analogue that inhibits viral RNA synthesis with a broad antiviral spectrum including filoviruses, paramyxoviruses, pneumoviruses, and coronaviruses. There is limited information on adverse effects. However, hypokalemia is a common reported side effect [52] and there was a patient who developed hypotension and bradycardia when this medication was used to treat Ebola [45]. The safety and efficacy of remdesivir for the treatment of COVID-19 are being evaluated in multiple ongoing Phase 3 clinical trials. In a recent randomized, double-blind, placebo-controlled, multicenter trial at ten hospitals in Hubei, China, conducted on adult patients admitted to hospital for severe COVID-19 disease, remdesivir was not associated with statistically significant clinical benefits. However, the numerical reduction in time to clinical improvement in those treated earlier requires confirmation in larger studies [52].

Tocilizumab an anti-IL-6R antibody is known for its potential efficacy in reducing inflammatory response including the cytokine storm contributing to ARDS and even death [4]. It is known to increase cholesterol levels, but there are conflicting reports on its effect on long term cardiac morbidity and mortality [53].

## Conclusion

Outbreaks of microbial illness in one country and because of ready access to international travel, remains a threat to global public health and global pandemics remain a persistent threat. COVID-19, primarily a respiratory disease, is a rapidly evolving pandemic with uncertain pathogenesis and clinical characteristics. But the associated extra-pulmonary manifestations and their prolonged consequences are frequently overlooked. Pre-existing cardiovascular disease or acute cardiac complications may contribute to adverse early clinical outcome. At the moment, there is no specific treatment for COVID-19, but multiple RCT are being conducted. New supportive therapies are being evaluated with promising results.

## Data Availability

not applicable.

## Abbreviations

SARS-CoV: Severe acute respiratory syndrome coronavirus
COVID-19: Coronavirus disease-19
RCT: Randomized controlled trials
MERS: Middle East respiratory syndrome
WHO: World Health Organization
RO: Reproductive number
ACE: Angiotensin converting enzyme
CVD: Cardiovascular disease
ICU: Intensive care unit
MR: Mortality rate
IL: Interleukin
GCSF: Granulocyte colony-stimulating factor
IP: Interferon
TNF: Tumor necrosis factor
rRT-PCR: Reverse transcriptase-polymerase chain reaction
NSAIDs: Non-steroidal anti-inflammatory drugs
ARDS: Acute respiratory distress syndrome
SIC: Sepsis-induced coagulopathy
